# Association of systemic anticholinergic medication use and accelerated decrease in lung function in older adults

**DOI:** 10.1038/s41598-024-54879-z

**Published:** 2024-02-22

**Authors:** Markus Svensson, Sölve Elmståhl, Johan Sanmartin Berglund, Aldana Rosso

**Affiliations:** 1 Division of Geriatric Medicine, Department of Clinical Sciences, Lund University, Jan Waldenströms Gata 35, 205 02 Malmö, Sweden; 2 Department of Health, Blekinge Institute of Technology, Karlskrona, Sweden

**Keywords:** Anticholinergics, FEV, Spirometry, Mixed models, Risk factors, Epidemiology

## Abstract

Older adults are frequently exposed to medicines with systemic anticholinergic properties, which are linked to increased risk of negative health outcomes. The association between systemic anticholinergics and lung function has not been reported. The aim of this study was to investigate if exposure to systemic anticholinergics influences lung function in older adults. Participants of the southernmost centres of the Swedish National study on Aging and Care (SNAC) were followed from 2001 to 2021. In total, 2936 subjects (2253 from Good Aging in Skåne and 683 from SNAC-B) were included. An extensive medical examination including spirometry assessments was performed during the study visits. The systemic anticholinergic burden was described using the anticholinergic cognitive burden scale. The effect of new use of systemic anticholinergics on the annual change in forced expiratory volume (FEV1s) was estimated using mixed models. During follow-up, 802 (27.3%) participants were exposed to at least one systemic anticholinergic medicine. On average, the FEV1s of participants without systemic anticholinergic exposure decreased 37.2 ml/year (95% CI [33.8; 40.6]) while participants with low and high exposure lose 47.2 ml/year (95% CI [42.4; 52.0]) and 43.7 ml/year (95% CI [25.4; 62.0]). A novel association between new use of medicines with systemic anticholinergic properties and accelerated decrease in lung function in older adults was found. The accelerated decrease is comparable to that observed in smokers. Studies are needed to further explore this potential side effect of systemic anticholinergics.

## Introduction

Approximately 60% of older adults have at least two or more chronic morbidities^1^. Prevalent conditions are hypertension, hyperlipidaemia, ischemic heart disease, diabetes, arthritis, heart failure, depression, and chronic kidney disease^2^. In consequence, this population is frequently exposed to several medications, and many of those have systemic anticholinergic properties^3^. Medicines with anticholinergic properties include antihistamines, antidepressants, antipsychotics, antispasmodics, muscarinic antagonists, and antiparkinsonian drugs. The anticholinergic effect of a drug can be due to primary anticholinergic activity (e.g., muscarinic antagonists) or secondary to the main therapeutic effect (e.g., antidepressants)^4^. The prevalence of prescriptions of these medicines depends on the clinical setting and has been estimated to be between 12 and 47%^5,6^. Older adults may have deficient renal and liver functions, and a diminished blood–brain barrier^7^, which may exacerbate the negative effects of anticholinergic medicines. There is evidence that exposure to systemic anticholinergics may increase the risk of mortality^8^, loss of physical functioning^9,10^, cognitive decline^9^, falls^10^, and dementia^11^. Anticholinergics primarily target the receptors of the neurotransmitter acetylcholine, which can inhibit the actions of the parasympathetic nervous system^4,12^. This may affect several parts of the body including the muscles located in the gastrointestinal track, urinary tract, and lungs. Thus, lung function, which decreases naturally with age due to reduced elasticity in lung tissue, debilitated muscles, and changes in the rib cage, could be affected by excessive exposure to these medicines. However, we are unaware of studies investigating this potential side effect in older adults. The aim of this work is to investigate whether exposure of medications with systemic anticholinergic properties influences lung function in older adults.

## Results

Baseline characteristics of the study participants are listed in Table 1. Overall, the participants from both centres were similar. The SNAC-B participants were slightly older, and a lower percentage had university education. The level of physical activity was similar in both cohorts despite GÅS participants reporting more former and current smokers. The disease burden was also comparable for both groups, although the percentage of COPD was higher for the GÅS cohort. All participants included in this study had an ACB score equal to 0 at baseline to investigate the effect of incident (new) use of systemic anticholinergics on lung function.

**Table 1:** Baseline characteristics of study participants.

Baseline characteristics		SNAC-B	GÅS	All participants
Number of participants (n)		683	2253	2936
Female (n, %)		375 (54.9)	1215 (53.9)	1590 (54.2)
Age (years), mean (SD) [min, max]		70.1 (9.7) [60.0,96.0]	68.5 (9.6) [59.2,94.0]	68.9 (9.6) [59.2,96.0]
Education level (n, %)	Primary school not completed	13 (1.9)	8 (0.4)	21 (0.7)
	Primary school completed	268 (39.2)	973 (43.2)	1241 (42.3)
	Secondary school	250 (36.6)	688 (30.5)	938 (31.9)
	University	123 (18.0)	579 (25.7)	702 (23.9)
	Missing	29 (4.2)	5 (0.2)	34 (1.2)
Former or current smoker (n, %)		331 (48.5)	1294 (57.4)	1625 (55.3)
	Missing	21 (3.1)	18 (0.8)	39 (1.3)
Physical activity level (n, %)	Sedentary	34 (5.0)	77 (3.4)	111 (3.8)
	Low intensity	373 (54.6)	1300 (57.7)	1673 (57.0)
	Moderate to high	208 (30.5)	851 (37.8)	1059 (36.1)
	Missing	68 (10.0)	25 (1.1)	93 (3.2)
COPD (n, %)		7 (1.0)	96 (4.3)	103 (3.5)
Cerebrovascular disease (n, %)		35 (5.1)	146 (6.5)	181 (6.2)
Diabetes (n, %)		33 (4.8)	139 (6.2)	172 (5.9)
Heart disease (n, %)		133 (19.5)	446 (19.8)	579 (19.7)
Hypertension (n, %)		218 (31.9)	577 (25.6)	795 (27.1)
Number of diseases, mean (SD) [min, max]		0.6 (0.8) [0.0,4.0]	0.6 (0.9) [0.0,4.0]	0.6 (0.9) [0.0,4.0]
Number of medicines, mean (SD) [min, max]		1.8 (1.8) [0.0,9.0]	1.4 (1.8) [0.0,13.0]	1.5 (1.8) [0.0,13.0]
Forced expiratory volume 1 s (L), mean (SD) [min, max]		2.4 (0.8) [0.0,5.0]	2.6 (0.9) [0.5,5.7]	2.6 (0.9) [0.0,5.7]

The mean follow-up time was 9 years (SD: 4) with a maximum of 19 years. Participants attended on average 3 study visits (SD: 1). The ten most frequently reported medicines during the study were acetylsalicylic acid, paracetamol, levothyroxine sodium, metoprolol, simvastatin, furosemide, cyanocobalamin, enalapril, amlodipine, and zopiclone. The most frequently reported medicines included in the ACB classification belong to the cardiovascular category and had ATC code C07, C01, and C03 (metoprolol [34%], furosemide [27%], atenolol [8%], isosorbide mononitrate [7%], digoxin [5%]). During follow-up, 802 (27.3%) participants were exposed to at least one medication with systemic anticholinergic properties included in the ACB scale. Of those, 186 (6.3%) discontinued use while the others remained exposed until lost to follow-up or end of the study, whichever came first. At most, there were 516 (17.6%) participants with ACB = 1 and 286 (9.7%) participants with ACB ≥ 2. The median number of medicines was 5 and 8 for ACB = 1 and ≥ 2, respectively.

The results of the mixed model implemented to investigate the association between the ACB score and the annual change from baseline of FEV1s are shown in Table 2. Since our model is optimized for the interpretation of the coefficients related to the anticholinergic burden, no conclusion is drawn regarding the effect of the other covariates on lung function^13^. On average, participants who developed ACB score = 1 during follow-up have an additional statistically significant decrease in FEV1s of 10.0 mL/year compared to participants who maintained ACB score = 0 (see Table 2). We also estimated an additional decrease of 6.5 mL/year in FEV1s for participants with ACB ≥ 2 relative to those non-exposed to anticholinergics, although the precision in this case is lower due to a limited sample size for the highly exposed group (see Table 2). The average adjusted predictions for the annual decrease in FEV1s for different subgroups and ACB categories were estimated using the mixed model (see Table 3). For all subgroups except COPD participants, it is observed that the exposure to systemic anticholinergics is associated with a larger decrease in lung function over time.

**Table 2:** Estimated mixed model coefficients for annual decline rate in FEV1s.

Variables	Estimate	Lower 95% CI	Upper 95% CI	P-value
Change FEV1s from baseline (mL/year) (Ref: ACB score = 0)				
ACB score = 1	− 10.0	− 15.9	− 4.1	0.001
ACB score ≥ 2	− 6.5	− 25.2	12.2	0.495
Baseline FEV1s (mL)	0.0	− 0.1	0.0	< 0.001
Age at baseline (Ref: 60–69 years)				
70–79 years	− 21.1	− 30.5	− 11.6	< 0.001
80–84 years	− 32.4	− 41.4	− 23.4	< 0.001
≥ 85 years	− 34.5	− 74.0	4.9	0.086
Number of diseases at baseline (Ref: 0)				
1	− 5.8	− 12.3	1.5	0.126
2	− 17.5	− 30.7	− 4.4	0.009
3 or more	− 21.7	− 40.7	− 2.8	0.024
Level of education (Ref: primary completed or not)				
Secondary	− 3.8	− 11.5	3.9	0.335
University	− 0.9	− 6.6	4.7	0.743
Physical activity level at baseline (Ref: moderate to high)				
Sedentary	7.1	− 34.9	49.0	0.741
Low intensity	− 3.7	− 10.3	2.8	0.264
Female (Ref: male)	− 38.1	− 49.5	− 26.7	< 0.001
Former or current smoker at baseline (Ref: non-smoker)	− 6.9	− 12.9	− 0.9	0.024
Constant	121.9	90.8	153.0	Not applicable
Number of visits	4143			
Number of participants	2503			

*CI* confidence interval.

**Table 3:** Estimated annual decline rate in FEV1s for different ACB categories and subgroups.

Subgroup		ACB score	Average estimated change in FEV1s from baseline (mL/Year)	Lower 95% CI	Upper 95% CI
Primary model	All participant (N = 2503)	0	− 37.2	− 40.6	− 33.8
		1	− 47.2	− 52.0	− 42.4
		≥ 2	− 43.5	− 62.0	− 25.4
Hypertension, heart disease or cerebrovascular disease at baseline or at follow-up	No (N = 1125)	0	− 35.4	− 39.8	− 31.0
		1	− 57.1	− 72.3	− 41.9
		≥ 2	− 28.7	− 50.1	− 7.3
	Yes (N = 1704)	0	− 38.5	− 43.5	− 33.4
		1	− 45.0	− 50.4	− 39.6
		≥ 2	− 47.3	− 68.3	− 26.3
COPD at baseline or at follow-up	No (N = 2340)	0	− 36.3	− 39.8	− 32.8
		1	− 47.7	− 52.9	− 42.6
		≥ 2	− 43.1	− 63.5	− 22.7
	Yes (N = 233)	0	− 51.8	− 61.5	− 42.1
		1	− 44.6	− 54.3	− 34.9
		≥ 2	− 45.6	− 61.4	− 29.8
Former and current smokers at baseline	No (N = 1090)	0	− 37.3	− 42.8	− 31.8
		1	− 42.7	− 51.8	− 33.6
		≥ 2	− 36.0	− 55.5	− 16.4
	Yes (N = 1413)	0	− 37.2	− 41.9	− 32.2
		1	− 45.0	− 51.0	− 38.9
		≥ 2	− 59.1	− 67.8	− 49.8

*CI* confidence interval.

Sensitivity analyses were performed to assess the robustness of the results regarding cohort and attrition effects (see Table 4). The results obtained only using the first follow-up visit were concordant with those obtained for the primary analysis. Notwithstanding the lack of precision due to reduced sample size, it is observed the decline rate in FEV1s estimated in the GÅS cohort is larger than that estimated for the SNAC-B cohort. These differences may be partially explained by the larger percentage of smokers and COPD clinically diagnosed participants in the GÅS cohort.

**Table 4:** Sensitivity analyses performed to assess cohort and attrition effects.

Analysis	ACB score	Average estimated change in FEV1s from baseline (mL/Year)	Lower 95% CI	Upper 95% CI
Primary model (N = 2503)	0	− 37.2	− 40.6	− 33.8
	1	− 47.2	− 52.0	− 42.4
	≥ 2	− 43.7	− 62.0	− 25.4
Only first follow up visit included (N = 2386)	0	− 35.6	− 40.2	− 31.0
	1	− 50.9	− 60.3	− 41.6
	≥ 2	− 28.8	− 69.4	11.8
GÅS cohort (N = 1957)	0	− 41.6	− 44.0	− 39.2
	1	− 48.2	− 53.2	− 43.2
	≥ 2	− 49.1	− 55.8	− 42.4
SNAC-B cohort (N = 546)	0	− 24.2	− 36.2	− 12.4
	1	− 43.9	− 57.4	− 30.4
	≥ 2	− 15.0	− 74.4	44.5

*CI* confidence interval.

## Discussion

In this study, we investigated the association between new use of medications with systemic anticholinergic properties and lung function in older adults. During follow-up, 27.3% of the study participants were exposed to medicines with systemic anticholinergic properties included in the ACB scale. The FEV1s decrease rate was estimated to 37.2, 47.2, and 43.7 mL/year for participants without exposure, with low exposure, and with moderate/high exposure to systemic anticholinergic medicines. Thus, our results indicate that exposure to systemic anticholinergic medicines is associated with an accelerated decline rate of the lung function in older adults. The FEV1s decline rate in people aged ≥ 65 years ranges from 17.7 to 46.4 mL/year with a median of 22.4 mL/year^14^. The observed additional decrease of 10 mL/year in FEV1s due to systemic anticholinergic exposure is similar to the estimated additional decrease seen in smokers compared to non-smokers and is thus not negligible^15^. Our results regarding a dose–response effect are inconclusive since the confidence intervals of the coefficients related to ACB score = 1 overlap with that for ACB score ≥ 2 (see Table 2), probably due to the limited sample size of the high exposed group and attrition and death of frail patients. We repeated the analyses in several subgroups (patients with cardiovascular disease, patients with COPD and smokers). In most cases, exposure to systemic anticholinergics was associated with an increase of FEV1s decline rate. Interestingly, patients diagnosed with COPD exposed to systemic anticholinergics seem to have a somewhat slower decrease rate compared to COPD patients without systemic anticholinergic treatment. While the backbone for COPD treatment is the inhalation of drugs with anticholinergic properties, those were not considered in this work since inhaled medications are excluded of the ACB classification. Anticholinergic treatments developed for respiratory diseases target three types of muscarinic receptors primarily involved in airway (patho)physiology: M1, M2, and M3^16^. Inhaled anticholinergic medications used to treat asthma and COPD (e.g., tiotropium) primarily bind to and block M1 and M3 receptors, which leads to decreased bronchoconstriction mediated via acetylcholine, and thus bronchodilation. However, the M2 receptor is an autoinhibitory receptor located on cholinergic nerve endings and acts as a negative feedback inhibitor of acetylcholine release from the nerve. Blocking this receptor leads to an increased release of acetylcholine, which leads to increased bronchoconstriction. If an anticholinergic agent has increased affinity/specificity for binding to M2 instead of M1 and M3, a paradoxical bronchoconstriction (i.e., decreased FEV1s) would occur^16^. Indeed, anticholinergic airway medications have been designed to have M1 + M3 receptor specificity over M2 receptor specificity to avoid this paradoxical effect^17^. However, systemic anticholinergics and drugs with secondary anticholinergic effects may not have been designed to have M1 + M3 specificity over M2 specificity. As stated, participants diagnosed with COPD get treated with inhaled anticholinergics specific to M1 + M3-receptors, possibly alleviating the effects of other anticholinergics on M2-receptors. Participants diagnosed with COPD also showed a slower decrease compared to baseline smokers. One explanation for this could be that smokers without a COPD diagnosis are not exposed to inhaled anticholinergics that would reduce the negative effects of systemic anticholinergics on lung function. In this study, we have not investigated the relative specificity of systemic anticholinergics on different muscarinic receptors. This makes our interpretation of mechanisms involving receptor affinity for the association of systemic anticholinergics with decreased lung function hypothetical. Despite a systematic search, we could not find any article reporting an accelerated decrease of lung function in older adults consuming medications with systemic anticholinergic properties.

Other pathways through which systemic anticholinergics may negatively affect lung function are not understood and there are several possible explanations. The long-term exposure to systemic anticholinergics may contribute to skeletal muscle weakness^18^, which may affect inspiratory and expiratory muscle strength^19^. Anticholinergics increases the level of sleepiness, changing the level of consciousness^20^, which may facilitate aspiration into the lungs^21^. Biological pathways for development of COPD include inflammation and accelerated aging which results in elastin degradation, endothelial dysfunction, and imbalances of protease and antiproteases. Proteases are involved in tissue remodelling, inflammation, and extracellular matrix degradation, promoting the pathological process of COPD^22^. A study of two systemic anticholinergic drugs, trihexyphenidyl used for the treatment of Parkinson disease and propiverine used for the treatment of overactive bladder, showed accelerated lipopolysaccharide induced neuronal inflammation, but also systemic inflammation in a mouse model pointing towards a possible pathway for lung function decline^23^.

As expected, participants exposed to medicines with anticholinergic properties also consumed several drugs, with a median number of 5 drugs for low (ACB score = 1), and 8 drugs for moderate/high anticholinergic exposure (ACB score ≥ 2). Polypharmacy increases the risk of drug-drug interactions and causes unexpected adverse events, which could partly explain our findings. It was not possible to disentangle the effect of consumption of anticholinergics from that of polypharmacy due to sample size limitations. At maximum anticholinergic exposure, only 25% and 10% of participants with ACB = 1 and ACB ≥ 2 did not have polypharmacy. In this study, most of the medicines with anticholinergic properties belong to the cardiovascular category. Cardiovascular disease (CVD) and lung disease frequently coexists in mid- and late-life, even among those without diagnosed lung disease^24^. Systemic inflammation appears to be an important underlying pathophysiological link. Furthermore, the MESA-COPD study of mild chronic lung disease noted an association to reduction in cardiac ventricular function that highlights the complex interplay between subclinical conditions^25^. Therefore, we cannot exclude a causal effect of CVD in the observed decrease in FEV1s. However, subgroup analyses indicate that our results are not driven by the inclusion of participants who were former or current smokers, with COPD or diagnosed with heart- and/or cerebrovascular disease since an increased decline in annual FEV1s due to exposure to systemic anticholinergic medicines is observed also in participants without those comorbidities.

## Strengths and limitations

This study has several strengths. We present information about the development over time of the lung function in older adults randomly selected from the general population. A well-developed medical protocol was implemented at each study visit, assuring consistency in the medical assessment. The decline rate in lung function could be estimated adequately due to the high number of re-examinations and the extensive follow-up period.

This study also has some limitations. Our results are based on new use of systemic anticholinergic medicines in an overall healthy older population. Therefore, the results reported may not apply to frail older adults or older subjects with a lifelong exposure to systemic anticholinergic medicines. Two cohorts with up to 19 years of follow-up were pooled. Sensitivity analyses were performed to investigate potential cohort effects and attrition bias. However, the potential influence of different drop-out reasons on the results could not be investigated due to limited information. Similar results as in the primary analysis were rendered, which alleviated our concern. The anticholinergic burden is typically described in epidemiological studies using classifications based on expert judgment. The choice of the anticholinergic scale may have impacted our results. Recent reviews^5,26^ concluded that the anticholinergic cognitive burden scale^27^ has good quality, is well-suited to describe the anticholinergic drug burden, and is associated with clinically negative outcomes in older adults. However, there are other scales than the ACB, which may give different results. We performed a sensitivity analysis using the ARS scale and obtained concordant results. This is in line with the work presented by Hanlon et al.^8^ who found that the anticholinergic drug burden is associated with adverse health outcomes regardless of the anticholinergic scale used. The ACB scale excludes topical, ophthalmic, otologic, and inhaled medications. In COPD and asthma patients, the ACB scale therefore probably underestimates the exposure level in this patient group. Most of the GÅS participants diagnosed with COPD are at early-stage of their disease and only a minority are treated with inhaled β2-agonist, muscarinic antagonists, corticosteroids, and/or combination therapy^28^. Therefore, we believe that the exclusion of inhaled medications in the ACB scale has a negligible effect in this study. We lack information about systemic anticholinergic exposure in-between the study visits. However, most of these medicines are typically prescribed to treat chronic conditions. Deprescribing is complex and not implemented in a systematic way in neither primary nor specialized healthcare^29^. Therefore, it is likely that participants were exposed to these medicines continuously during the study period. Medicine compliance cannot be granted. Participants brought their medicines to the study visits. Information about medicine consumption of participants living in nursing homes was obtained from their nurses. Thus, we believe that the reported medicines reflect actual consumption. Despite collecting extensive information about health status and lifestyle, unmeasured confounding remains. Since lung function is negatively affected by cardiovascular disease, confounding by indication is possible.

In conclusion, a novel association between exposure of medications with systemic anticholinergic properties and accelerated decrease in lung function in older adults was found. The estimated additional loss of 10 mL/year is comparable to the accelerated decrease in FEV1s that has been attributed to smoking. Additional studies are needed to further explore this potential side effect of systemic anticholinergics.

## Methods

### Study population and design

**Figure 1:**
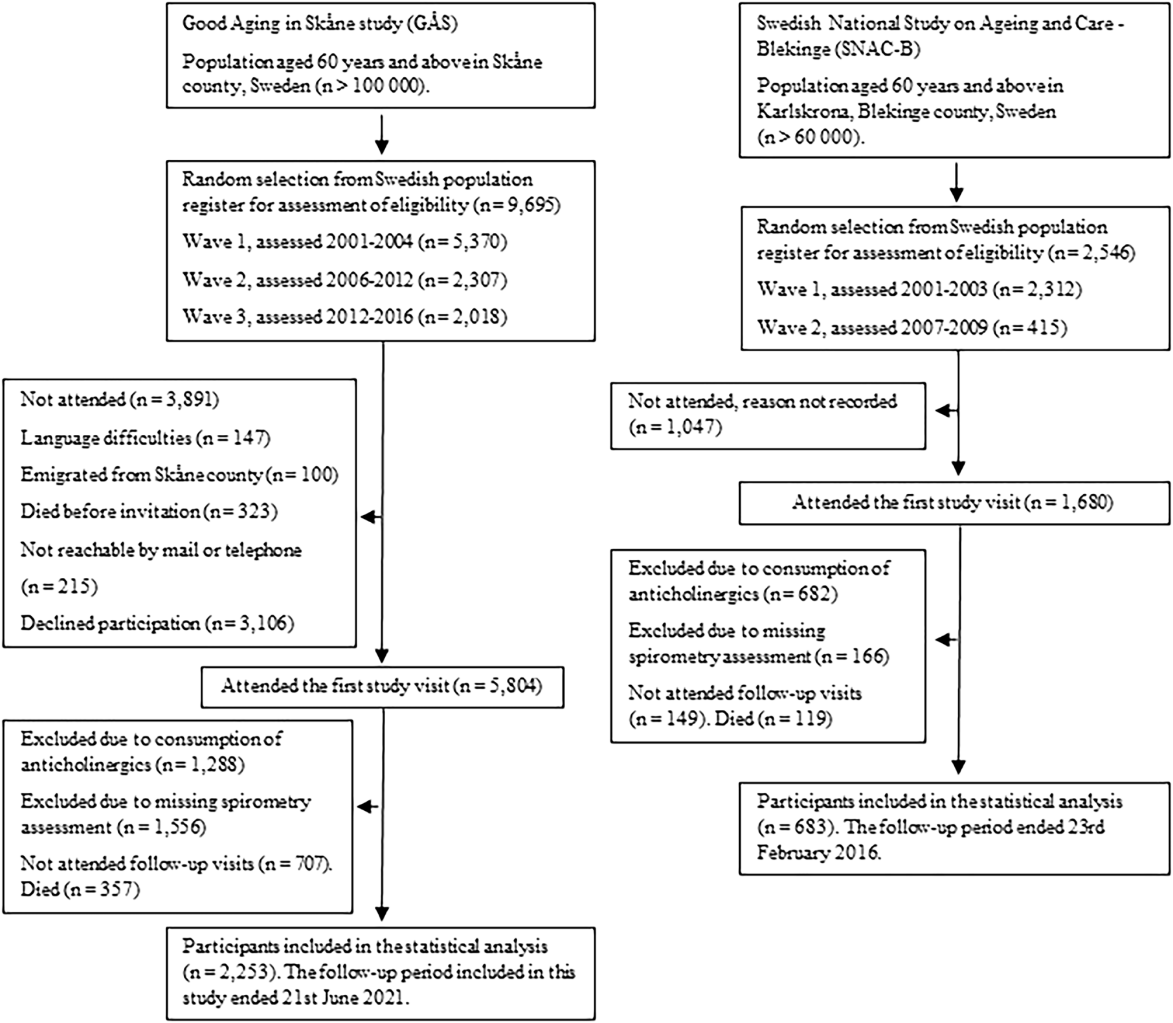
Flowchart indicating the inclusion of participants for GÅS and SNAC-B.

The Swedish national Study on Aging and Care (SNAC) is an ongoing population-based, multicentre cohort study, which started enrolment of older adults in 2001^30^. The data presented here were collected at the centres located in the south of Sweden, Skåne (Good Aging in Skåne, GÅS) and Blekinge (SNAC-B). The study design has been described elsewhere^31,32^. Briefly, subjects aged 60 years or more living in the cities of Eslöv, Hässleholm, Malmö, Osby, Ystad (GÅS), or Karlskrona (SNAC-B) are randomly selected from the Swedish population register and encouraged to participate in the study. Participants are offered a thorough physical, medical, and psychological examination and invited to attend to follow-up examinations at regular intervals every three (≥ 78 years of age) to six (< 78 years of age) years until death. To encourage participation of frail adults, the study team performs home visits. However, participants may receive a shorter examination if they are not able or willing to perform a full examination. Currently, two and three waves have been fully recruited at SNAC-B and GÅS, respectively, with an initial participation rate of approximately 60%. As shown in Fig. 1, data from 2936 participants examined between 13th Feb 2001 and 21st Jun 2021, and 26th March 2001 and 23rd Feb 2016 were retrieved from GÅS and SNAC-B, respectively. This study focuses only on the effect of new use of systemic anticholinergics to minimize bias^33^. Consequently, participants exposed to any systemic anticholinergic medicine included in the anticholinergic cognitive burden scale at baseline were excluded.

### Ethics

The SNAC study is conducted according to the Declaration of Helsinki and Good Clinical Practice guidelines. The study was approved by the Lund University Ethics Review Board (LU 744-00). All participants provide written informed consent.

### Sociodemographics, identification of morbidities and medicine consumption

Sociodemographic variables included age, sex, and level of formal education. The level of physical activity was self-reported. Chronic diseases of interest for this study were: hypertension, heart disease, cerebrovascular disease, diabetes type 1 and 2, and chronic pulmonary obstructive disease (COPD). Cerebrovascular disease included cerebral infarction, cerebral haemorrhage, occlusion and stenosis of precerebral or cerebral arteries, and/or transient cerebral ischemia. Heart disease included acute myocardial infarction, ischemic heart disease, presence of cardiac and vascular implants, heart failure, and atrial fibrillation or other arrhythmias. COPD also included emphysema and chronic bronchitis. These morbidities were identified during the medical examination and by retrieving medical records. The disease burden was summarized by the number of chronic diseases diagnosed per participant, which is a frequently implemented approach^34,35^. Information about prescribed medicines was self-reported to the study nurse (SNAC-B) or the study physician (GÅS) and discrepancies with medical records were discussed with the participants.

### Spirometry assessments

Trained research staff performed all spirometry measurements using a Vitalograph 2120 spirometer (Vitalograph Ltd, Buckingham, UK) according to the American Thoracic Society guidelines^36^. Several attempts were made, and if the subjects were able to demonstrate a satisfactory technique, the forced expiratory volume in one-second (FEV1s) from the best three attempts was recorded. Bronchodilators were not administrated during the first wave baseline visit in the GÅS study. Subjects received 1.0 mg of β2-receptor agonist terbutaline 10 min prior to the spirometry at all other visits.

### Anticholinergic burden

We defined the systemic anticholinergic burden using the anticholinergic cognitive burden scale (ACB) developed by Boustani et al.^27^, which has been implemented in several epidemiological studies^8,11^. This scale was developed specifically to investigate cognitive side effects and includes 88 medicines. Topical, ophthalmic, otologic, and inhaled medication preparations were excluded. The anticholinergic activity of each medicine is classified as 0 (no anticholinergic effect), 1 (possible anticholinergic effect), 2 or 3 (established and clinically relevant cognitive anticholinergic effects). The sum of the scores of the medicines taken by the individual determines the ACB total score, e.g., the anticholinergic burden. The medicines included in the ACB scale are listed in the Supplementary material Table S1.

### Statistical analysis

The annual change in forced expiratory volume in the first second was defined as the difference in FEV1s between two consecutive study visits, divided by the time passed between the visits. This model assumes that the annual decline rate is constant over time. A mixed model for repeated measures with random intercept (participants) was implemented. Using a directed acyclic graph (DAG), we identified which factors were required in the model to estimate the total effect of systemic anticholinergic drug burden on lung function (Fig. S1). The variables age, sex, smoking status, formal education level, physical activity level, and number of chronic diseases (at baseline) were thus included to mitigate confounding. The FEV1s baseline value was included in the model to improve precision. The anticholinergic burden using the ACB scale was modelled as a time-dependent covariate and the score was updated at each visit to reflect the current medication list. Confidence intervals were calculated using robust standard errors. Goodness of the fit was assessed visually using residual plots. We also calculated average adjusted predictions (AAP) to estimate the annual FEV1s change for different ACB values. First, the mixed model calculates the predicted FEV1s decline for each subject. Second, these probabilities are averaged, and an estimate is obtained for the study population. The statistical analyses were performed using Stata IC 17.0 software (StataCorp LLC, Texas, USA), and Python 3.8.5 (Python Software Foundation).

### Subgroup analyses

Mixed models were calculated for participants diagnosed with hypertension, heart disease, or cerebrovascular disease; former and current smokers; or diagnosed with COPD.

### Sensitivity analyses

Data from two SNAC centres were pooled since the SNAC study was designed to allowing pooling of data from different centres. However, there are minor differences in the medical examination, wording of some questions, and study population, which may affect the comparability of the cohorts. Cohort effects were investigated by performing the analyses separately for SNAC-B and GÅS. Attrition is inevitable in cohort studies involving older adults. The GÅS and SNAC-B cohorts have 19 and 12 years of follow-up, respectively. Due to the long follow-up period, frail participants are less sampled, and differential attrition occurs. In addition, our statistical model assumes that the decline rate is constant over time, which is correct only when two visits are considered. Thus, we conducted a sensitivity analysis restricted to the first follow-up visit. Currently, there is no consensus on which anticholinergic burden instrument provides the most relevant clinical information^11^. Therefore, we conducted a sensitivity analysis using the anticholinergic risk scale (ARS) developed by Rudolph et al.^37^. This analysis is presented in the electronic supplementary material (Fig. S2 and Table S2).

### Supplementary Information

Supplementary Information.

## Data Availability

Data are accessible on request (https://neardb.near-aging.se/study/gas-snac-s). Requests can also be sent to ole.larsen@med.lu.se or PI solve.elmstahl@med.lu.se.
