# Supplementary material for: Association of systemic anticholinergic medication use and accelerated decrease in lung function in older adults
Source: Sci Rep. 2024 Feb 22;14:4362. doi: 10.1038/s41598-024-54879-z (PMC10883995; doi:10.1038/s41598-024-54879-z)
Supplement: Supplementary file 1 — Supplementary Information. [file 41598_2024_54879_MOESM1_ESM.docx]

**Association of systemic anticholinergic medication use and accelerated decrease in lung function in older adults**

Markus Svensson^1^, MD, Sölve Elmståhl^1^, MD, PhD, Prof., Johan Sanmartin Berglund^2^, MD, PhD, Prof, Aldana Rosso^1^, PhD

^1^ Division of Geriatric Medicine, Department of Clinical Sciences, Lund University, Malmö, Sweden

^2^ Blekinge Institute of Technology, Department of Health, Karlskrona, Sweden

Corresponding Author: Markus Svensson

Division of Geriatric Medicine, Department of Clinical Sciences in Malmö, Lund University

Jan Waldenströms gata 35, 205 02 Malmö, Sweden

Tel: +46 766 44 44 27

E-mail:  [markus.svensson@med.lu.se](mailto:%20markus.svensson@med.lu.se)

**Anticholinergic cognitive burden scale**

The anticholinergic cognitive burden (ACB) scale was developed by Boustani et al. (1). There are 88 medicines included in this scale, which are listed below.

| **Pharmacologic group** | **ATC code** | **Substance** | **Points** |
| --- | --- | --- | --- |
| H1-antihistamines | R06AD01 | Alimemazine | 1 |
| Drugs for gastrointestinal disorders | A03AX08 | Alverine | 1 |
| Anxiolytics or hypnotics | N05BA12 | Alprazolam | 1 |
| Drugs related to cardiovascular system | C07AB03 | Atenolol | 1 |
| Antidepressants | N06AX12 | Bupropion | 1 |
| Drugs related to cardiovascular system | C09AA01 | Captopril | 1 |
| Drugs related to cardiovascular system | C03BA04 | Chlortalidone | 1 |
| Drugs for gastrointestinal disorders | A02BA01 | Cimetidine | 1 |
| Drugs for gastrointestinal disorders | A02BA02 | Ranitidine | 1 |
| Anxiolytics or hypnotics | N05BA05 | Clorazepate | 1 |
| Other | R05DA04 | Codeine | 1 |
| Drugs related to cardiovascular system | B01AA03 | [Warfarin](https://www.whocc.no/atc_ddd_index/?code=B01AA03) | 1 |
| Other | M04AC01 | Colchicine | 1 |
| Anxiolytics or hypnotics | N05BA01 | Diazepam | 1 |
| Drugs related to cardiovascular system | C01AA05 | Digoxin | 1 |
| Drugs related to cardiovascular system | B01AC07 | [Dipyridamole](https://www.whocc.no/atc_ddd_index/?code=B01AC07) | 1 |
| Drugs related to cardiovascular system | C01BA03 | [Disopyramide](https://www.whocc.no/atc_ddd_index/?code=C01BA03) | 1 |
| Other | N01AH01 | [Fentanyl](https://www.whocc.no/atc_ddd_index/?code=N01AH01) | 1 |
| Drugs related to cardiovascular system | C03CA01 | [Furosemide](https://www.whocc.no/atc_ddd_index/?code=C03CA01) | 1 |
| Antidepressants | [N06AB08](https://www.whocc.no/atc_ddd_index/?code=N06AB08) | Fluvoxamine | 1 |
| Antipsychotics, normothymics and antiepileptics | [N05AD01](https://www.whocc.no/atc_ddd_index/?code=N05AD01) | Haloperidol | 1 |
| Drugs related to cardiovascular system | C02DB02 | [Hydralazine](https://www.whocc.no/atc_ddd_index/?code=C02DB02) | 1 |
| Corticoids | H02AB09 | [Hydrocortisone](https://www.whocc.no/atc_ddd_index/?code=H02AB09) | 1 |
| Drugs related to cardiovascular system | C01DA08 | [Isosorbide dinitrate](https://www.whocc.no/atc_ddd_index/?code=C01DA08) | 1 |
| Drugs related to cardiovascular system | C01DA14 | [Isosorbide mononitrate](https://www.whocc.no/atc_ddd_index/?code=C01DA14) | 1 |
| Drugs for gastrointestinal disorders | A07DA03 | Loperamide | 1 |
| Drugs related to cardiovascular system | C07AB02 | [Metoprolol](https://www.whocc.no/atc_ddd_index/?code=C07AB02) | 1 |
| Analgesics | N02AA01 | [Morphine](https://www.whocc.no/atc_ddd_index/?code=N02AA01) | 1 |
| Drugs related to cardiovascular system | C08CA05 | [Nifedipine](https://www.whocc.no/atc_ddd_index/?code=C08CA05) | 1 |
| Corticoids | H02AB07 | [Prednisone](https://www.whocc.no/atc_ddd_index/?code=H02AB07) | 1 |
| Drugs related to cardiovascular system | C01BA01 | [Quinidine](https://www.whocc.no/atc_ddd_index/?code=C01BA01) | 1 |
| Antipsychotics, normothymics and antiepileptics | [N05AX08](https://www.whocc.no/atc_ddd_index/?code=N05AX08) | Risperidone | 1 |
| Antiasthmatic drugs | R03DA04 | [Theophylline](https://www.whocc.no/atc_ddd_index/?code=R03DA04) | 1 |
| Antidepressants | [N06AX05](https://www.whocc.no/atc_ddd_index/?code=N06AX05) | Trazodone | 1 |
| Drugs related to cardiovascular system | [C03DB02](https://www.whocc.no/atc_ddd_index/?code=C03DB02) | Triamterene | 1 |
| Antiparkinsonian | N04BB01 | [Amantadine](https://www.whocc.no/atc_ddd_index/?code=N04BB01) | 2 |
| Drugs for gastrointestinal disorders | A03BA04 | [Belladonna total alkaloids](https://www.whocc.no/atc_ddd_index/?code=A03BA04) | 2 |
| Antipsychotics, normothymics and antiepileptics | N03AF01 | [Carbamazepine](https://www.whocc.no/atc_ddd_index/?code=N03AF01) | 2 |
| Muscle relaxants | [M03BX08](https://www.whocc.no/atc_ddd_index/?code=M03BX08) | Cyclobenzaprine | 2 |
| H1-antihistamines | [R06AX02](https://www.whocc.no/atc_ddd_index/?code=R06AX02) | Cyproheptadine | 2 |
| Antipsychotics, normothymics and antiepileptics | N05AH01 | [Loxapine](https://www.whocc.no/atc_ddd_index/?code=N05AH01) | 2 |
| Analgesics | N02AB02 | [Pethidine](https://www.whocc.no/atc_ddd_index/?code=N02AB02) | 2 |
| Antipsychotics, normothymics and antiepileptics | N05AA02 | [Levomepromazine](https://www.whocc.no/atc_ddd_index/?code=N05AA02) | 2 |
| Antipsychotics, normothymics and antiepileptics | N05AE02 | [Molindone](https://www.whocc.no/atc_ddd_index/?code=N05AE02) | 2 |
| Antipsychotics, normothymics and antiepileptics | N03AF02 | [Oxcarbazepine](https://www.whocc.no/atc_ddd_index/?code=N03AF02) | 2 |
| Antipsychotics, normothymics and antiepileptics | [N05AG02](https://www.whocc.no/atc_ddd_index/?code=N05AG02) | Pimozide | 2 |
| Antidepressants | N06AA09 | [Amitriptyline](https://www.whocc.no/atc_ddd_index/?code=N06AA09) | 3 |
| Antidepressants | N06AA17 | [Amoxapine](https://www.whocc.no/atc_ddd_index/?code=N06AA17) | 3 |
| Drugs for gastrointestinal disorders | A03BA01 | [Atropine](https://www.whocc.no/atc_ddd_index/?code=A03BA01) | 3 |
| Antiparkinsonian | N04AC01 | [Benzatropine](https://www.whocc.no/atc_ddd_index/?code=N04AC01) | 3 |
| H1-antihistamines | R06AB01 | [Brompheniramine](https://www.whocc.no/atc_ddd_index/?code=R06AB01) | 3 |
| H1-antihistamines | [R06AA08](https://www.whocc.no/atc_ddd_index/?code=R06AA08) | Carbinoxamine | 3 |
| H1-antihistamines | R06AB04 | [Chlorphenamine](https://www.whocc.no/atc_ddd_index/?code=R06AB04) | 3 |
| Antipsychotics, normothymics and antiepileptics | [N05AA01](https://www.whocc.no/atc_ddd_index/?code=N05AA01) | Chlorpromazine | 3 |
| H1-antihistamines | R06AA04 | [Clemastine](https://www.whocc.no/atc_ddd_index/?code=R06AA04) | 3 |
| Antidepressants | N06AA04 | [Clomipramine](https://www.whocc.no/atc_ddd_index/?code=N06AA04) | 3 |
| Antipsychotics, normothymics and antiepileptics | N05AH02 | [Clozapine](https://www.whocc.no/atc_ddd_index/?code=N05AH02) | 3 |
| Urinary antispasmodics | [G04BD10](https://www.whocc.no/atc_ddd_index/?code=G04BD10) | Darifenacin | 3 |
| Antidepressants | N06AA01 | [Desipramine](https://www.whocc.no/atc_ddd_index/?code=N06AA01) | 3 |
| Drugs for gastrointestinal disorders | A03AA07 | [Dicycloverine](https://www.whocc.no/atc_ddd_index/?code=A03AA07) | 3 |
| H1-antihistamines | R06AA11 | [Dimenhydrinate](https://www.whocc.no/atc_ddd_index/?code=R06AA11) | 3 |
| H1-antihistamines | R06AA02 | [Diphenhydramine](https://www.whocc.no/atc_ddd_index/?code=R06AA02) | 3 |
| Antidepressants | N06AA12 | [Doxepin](https://www.whocc.no/atc_ddd_index/?code=N06AA12) | 3 |
| Urinary antispasmodics | G04BD02 | [Flavoxate](https://www.whocc.no/atc_ddd_index/?code=G04BD02) | 3 |
| Anxiolytics or hypnotics | N05BB01 | Hydroxyzine | 3 |
| Drugs for gastrointestinal disorders | A03BA03 | [Hyoscyamine](https://www.whocc.no/atc_ddd_index/?code=A03BA03) | 3 |
| Antidepressants | N06AA02 | [Imipramine](https://www.whocc.no/atc_ddd_index/?code=N06AA02) | 3 |
| H1-antihistamines | R06AE05 | [Meclozine](https://www.whocc.no/atc_ddd_index/?code=R06AE05) | 3 |
| Antidepressants | N06AA10 | [Nortriptyline](https://www.whocc.no/atc_ddd_index/?code=N06AA10) | 3 |
| Antipsychotics, normothymics and antiepileptics | N05AH03 | [Olanzapine](https://www.whocc.no/atc_ddd_index/?code=N05AH03) | 3 |
| Antiparkinsonian | N04AB02 | [Orphenadrine (chloride)](https://www.whocc.no/atc_ddd_index/?code=N04AB02) | 3 |
| Urinary antispasmodics | G04BD04 | [Oxybutynin](https://www.whocc.no/atc_ddd_index/?code=G04BD04) | 3 |
| Antidepressants | N06AB05 | [Paroxetine](https://www.whocc.no/atc_ddd_index/?code=N06AB05) | 3 |
| Antipsychotics, normothymics and antiepileptics | N05AB03 | [Perphenazine](https://www.whocc.no/atc_ddd_index/?code=N05AB03) | 3 |
| Antiparkinsonian | [N04AA04](https://www.whocc.no/atc_ddd_index/?code=N04AA04) | Procyclidine | 3 |
| Antipsychotics, normothymics and antiepileptics | N05AA03 | [Promazine](https://www.whocc.no/atc_ddd_index/?code=N05AA03) | 3 |
| H1-antihistamines | R06AD02 | [Promethazine](https://www.whocc.no/atc_ddd_index/?code=R06AD02) | 3 |
| Drugs for gastrointestinal disorders | A03AB05 | [Propantheline](https://www.whocc.no/atc_ddd_index/?code=A03AB05) | 3 |
| Antiasthmatic drugs | R03DA12 | [Mepyramine theophyllinacetate](https://www.whocc.no/atc_ddd_index/?code=R03DA12) | 3 |
| Antipsychotics, normothymics and antiepileptics | [N05AH04](https://www.whocc.no/atc_ddd_index/?code=N05AH04) | Quetiapine | 3 |
| Other | A04AD01 | [Scopolamine](https://www.whocc.no/atc_ddd_index/?code=A04AD01) | 3 |
| Antipsychotics, normothymics and antiepileptics | [N05AC02](https://www.whocc.no/atc_ddd_index/?code=N05AC02) | Thioridazine | 3 |
| Urinary antispasmodics | [G04BD07](https://www.whocc.no/atc_ddd_index/?code=G04BD07) | Tolterodine | 3 |
| Antipsychotics, normothymics and antiepileptics | N05AB06 | [Trifluoperazine](https://www.whocc.no/atc_ddd_index/?code=N05AB06) | 3 |
| Antiparkinsonian | N04AA01 | [Trihexyphenidyl](https://www.whocc.no/atc_ddd_index/?code=N04AA01) | 3 |
| Antidepressants | N06AA06 | [Trimipramine](https://www.whocc.no/atc_ddd_index/?code=N06AA06) | 3 |

**Table S1:** Medicines included in the anticholinergic cognitive burden scale

**Directed acyclic graph (DAG)**

Using a directed acyclic graph (DAG), we identified which factors were required in the model to estimate the total effect of systemic anticholinergic drug burden on lung function. The variables age, sex, smoking status, formal education level, physical activity level, and number of chronic diseases (at baseline) were thus included to mitigate confounding.


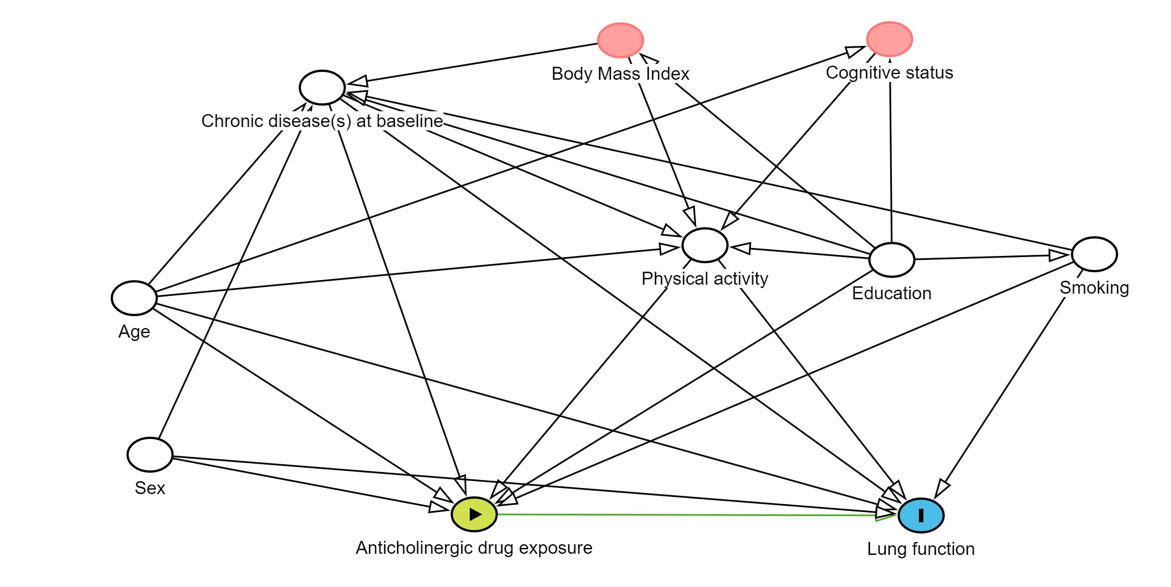


**Figure S1.** Directed acyclic graph (DAG) identifying which factors were required in the model to estimate the total effect of systemic anticholinergic drug burden on lung function.

**Sensitivity analysis using the anticholinergic risk scale**

Rudolph et al developed the Anticholinergic Risk Scale (ARS) to predict the risk of anticholinergic adverse effects in the geriatric population (2). The scale ranks medications for anticholinergic potential on a 3-point scale (0 no or low potential,3 high anticholinergic potential). The score is calculated as the sum of the points for each person. The scale is based on expert review of a list of 500 medication. Topical, ophthalmic, otologic, and inhaled

The primary analysis was repeated using the ARS scale to describe the anticholinergic burden.

The sample included in this analysis is shown in Figure S2. As in the primary analysis, participants with an ARS score ≥ 1 at baseline were excluded.


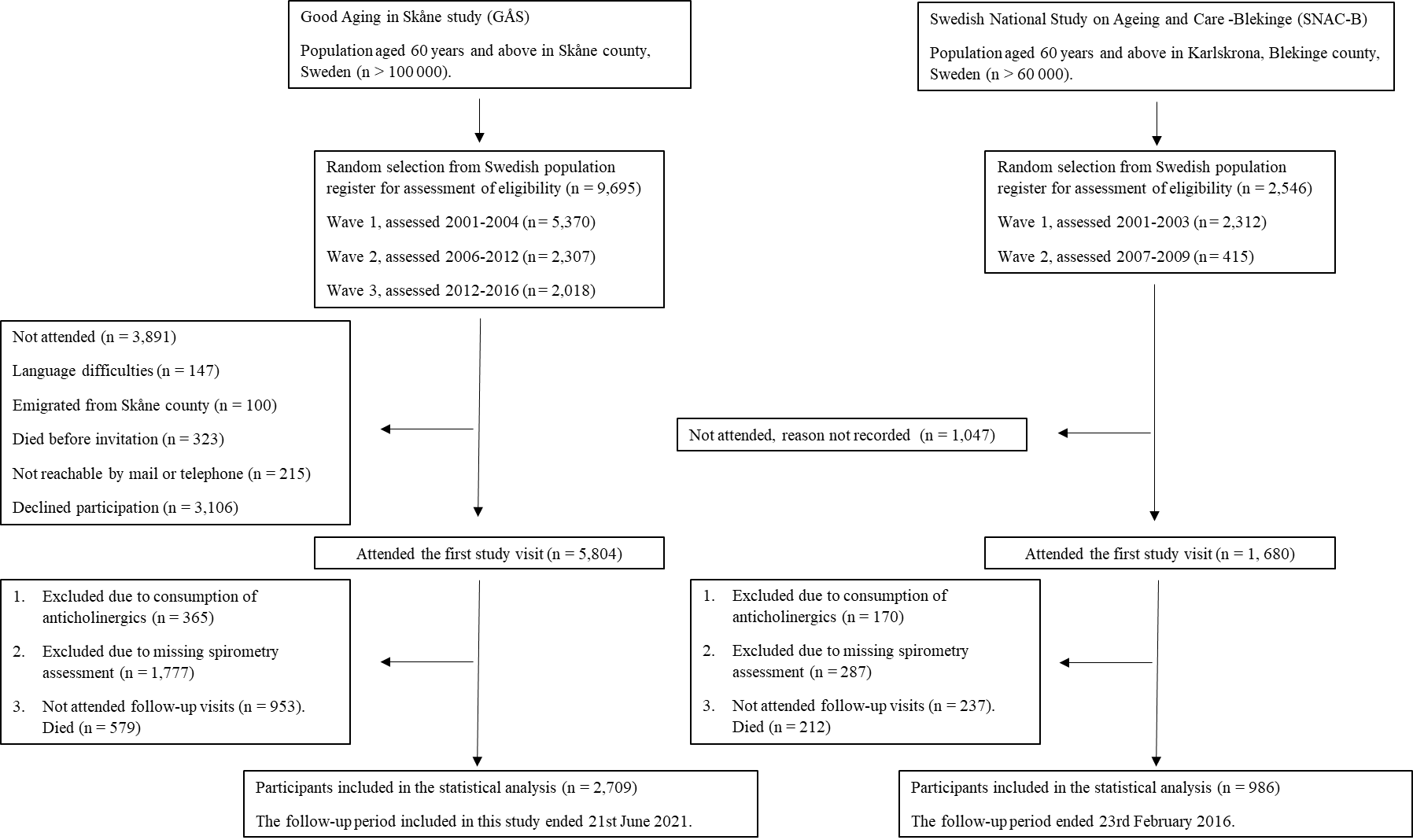


**Figure S2.** Flowchart indicating the inclusion of participants for GÅS and SNAC-B for the sensitivity analysis using the anticholinergic risk scale.

A mixed model using the same explanatory variables as the primary analysis was implemented (age, sex, smoking status, formal education level, physical activity level, number of chronic diseases at baseline). The FEV1s baseline value was included in the model to improve precision. The anticholinergic burden using the ARS scale was modelled as a time-dependent covariate. The ARS score was dichotomized as 0 no anticholinergic risk, 1 or more any anticholinergic risk.

The estimated annual change in FEV1s is shown in Table S2. The results of the sensitivity analysis are concordant with those obtained in the primary analysis

| **Analysis** | **Score** | **Average estimated change in FEV1s from baseline (mL/Year)** | **Lower 95 % CI** | **Upper 95 % CI** |
| --- | --- | --- | --- | --- |
| Primary model with ACB scale (N= 2,503) | 0 | -37.2 | -40.6 | -33.8 |
|  | 1 | -47.2 | -52.0 | -42.4 |
|  | 2 | -43.7 | -62.0 | -25.4 |
| Sensitivity analysis with ARS scale  (N=2,338) | 0 | -42.1 | -44.1 | -40.1 |
|  | ≥1 | -50.5 | -58.1 | -42.9 |

**Table S2:** Results of the sensitivity analysis using the anticholinergic risk scale

**References**

1. Boustani M, Campbell N, Munger S, Maidment I, Fox C. Impact of anticholinergics on the aging brain: a review and practical application. Aging Health. 2008;4(3):311-20 <https://doi.org/10.2217/1745509x.4.3.311>.

2. Rudolph JL, Salow MJ, Angelini MC, McGlinchey RE. The Anticholinergic Risk Scale and Anticholinergic Adverse Effects in Older Persons. Archives of Internal Medicine. 2008;168(5):508-13 <https://doi.org/10.1001/archinternmed.2007.106>.
